# Polymorphisms of Mouse Apolipoprotein A-II Alter Its Physical and Functional Nature

**DOI:** 10.1371/journal.pone.0088705

**Published:** 2014-02-10

**Authors:** Timothy J. Sontag, Catherine A. Reardon

**Affiliations:** Department of Pathology, University of Chicago, Chicago, Illinois, United States of America; Harvard Medical School, United States of America

## Abstract

ApoA-II is the second most abundant protein on HDL making up ∼20% of the total protein but its functions have still only been partially characterized. Recent methodological improvements have allowed for the recombinant expression and characterization of human apoA-II which shares only 55% sequence homology with murine apoA-II. Here we describe the purification of the two most common polymorphic variants of apoA-II found in inbred mouse strains, differing at 3 amino acid sites. C57BL/6 mice having variant apoA-II^a^ have lower plasma HDL levels than FVB/N mice that have variant apoA-II^b^. Characterization of the helical structure of these two variants reveals a more alpha-helical structure for the FVB/N apoA-II. These changes do not alter the lipid or HDL binding of the two apoA-II variants, but significantly increase the ability of the FVB/N variant to promote both ABCA1 and ABCG1 mediated cellular cholesterol efflux. These differences may be differentially altering plasma HDL apoA-II levels. In vivo, neither C57 nor FVB apoA-II protein levels are affected by the absence of apoE, while an apoE/apoA-I double deficiency results in a 50% decrease of plasma FVB apoA-II but results in undetectable levels of C57 apoA-II in the plasma. FVB apoA-II is able to form an HDL particle in the absence of apoE or apoA-I.

## Introduction

Cardiovascular disease including atherosclerosis continues to be a leading cause of morbidity and mortality. Epidemiologic evidence along with experimental data has implicated High Density Lipoprotein (HDL) as a negative risk factor for cardiovascular disease [1]. The protein component of HDL is primarily made up of apolipoproteins (apo) A-I and A-II, with apoA-I comprising 70–80%, and apoA-II 20% [2]. ApoA-I has been widely studied with numerous studies indicating an athero-protective role for apoA-I [3]–[7]. Less is known about the role of apoA-II. In humans there are conflicting results, with some studies showing an inverse correlation between plasma apoA-II levels and coronary atherosclerosis and future disease risk [8], [9]. However, human apoA-II deficiency does not impart increased risk of atherosclerotic disease [10]. Murine studies are similarly equivocal. ApoA-II overexpression increases aortic lesion size despite increased plasma HDL levels, while knocking out apoA-II increases the atherogenic properties of murine HDL [11], [12]. Murine apoA-II shares only a 55% sequence homology with human apoA-II and exists as a monomer, lacking the cysteine residue in human apoA-II [13]. Several mouse apoA-II sequence variants have been identified with apoA-II^a^ and apoA-II^b^ being among the most common of the variants, differing at 3 amino acid sites in the mature apoprotein (D20E, M26V, A28V, respectively) [14]. Among inbred mouse strains the C57BL/6 (C57), possessing the apoA-II^a^ variant, is highly athero-susceptible while the FVB/N (FVB) expressing the apoA-II^b^ is athero-resistant [15], [16]. The FVB strain has double the plasma apoA-II concentration of the C57 as well as double the HDL cholesterol [17], not surprising as murine plasma apoA-II levels are highly correlated with HDL cholesterol levels and with the apoA-II polymorphisms that the FVB mouse possesses [18], [19]. Indeed, these FVB polymorphisms are associated with similar apoA-II mRNA expression levels as the C57 variant, but increased protein synthesis, leading to increased plasma levels [19]. It is not known however if these polymorphisms also alter the function of the mature apoA-II protein or its HDL interaction. Wang and colleagues have demonstrated that the absence of apoA-I results in a redistribution of C57 apoA-II to a larger sized HDL particle [20]. However, the interaction of FVB apoA-II with HDL in the absence of apoA-I is not known. One of the roadblocks in studying the functional aspects of apoA-II has been that the lipophilic nature of apoA-II has made it difficult to achieve high yields of the protein using recombinant expression. Recently, Smith and colleagues have developed a novel method for high yield expression of human apoA-II in *E. coli* [21]. Using a modified version of this protocol, we have here expressed recombinant murine apoA-II of both the C57 and FVB isoforms and determined that they differ in certain aspects of their lipid and HDL interaction. Additionally we demonstrate a redistribution of the apoA-II forms in apoA-I/apoE double knockout mice of both strain backgrounds.

## Materials and Methods

### Mice

Female Wild type C57BL/6J, FVB/NJ and C57BL/6J *Apoe^−/−^* (CE) and *Apoa1^−/−^* mice were purchased from Jackson Laboratory, Bar Harbor, ME and crossed to yield homozygous *Apoe^−/−^/Apoa1^−/−^* double knockout mice on the C57 background (CEA). FVBN/J *Apoe^−/−^* mice (FE) were a generous gift from Dr. Jan Breslow (Rockefeller University, New York, NY) [15]. FE mice were crossed with CEA mice and the *Apoa1+/−* heterozygous progeny were backcrossed 10 generations into the FE background at which point the *Apoa1+/−* heterozygous mice were crossed to yield *Apoe^−/−^/Apoa1^−/−^* double knockout mice on the FVB background (FEA). The mice were bred in a specific pathogen free facility. Female mice at 8–10 weeks of age were used for all experiments and maintained on chow diet #7913 (6.2% fat) from Harlan Teklad (Indianapolis, IN). All mouse studies and euthanasia were performed in accordance with *The Guide for the Care and Use of Laboratory Animals* and National Institutes of Health guidelines and approved by the University of Chicago Institutional Animal Care and Use Committee.

### Mouse apoA-II expression and purification

**Figure 1:**
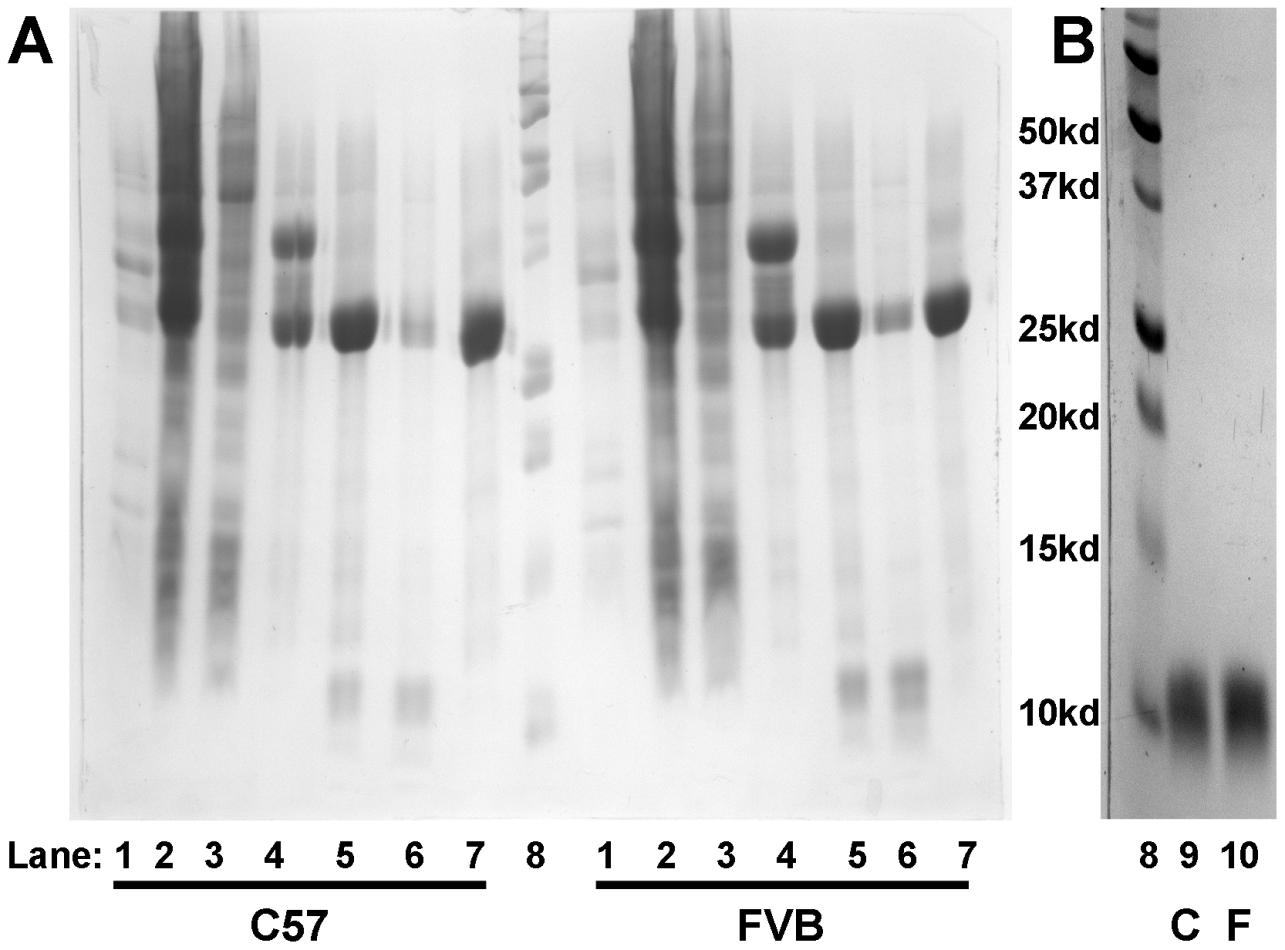
Purification of recombinant mouse apoA-II. (**A**) Expression and cleavage of apoA-II-Intein-Chitin binding domain fusion protein. Lanes 1: Post lysis pellet, 2: Post lysis supernatant, 3: Post Chitin unbound elution, 4: Bound fusion protein, 5: Post DTT cleave/pre elution Chitin bead, 6: Post DTT cleave eluted protein, 7: Post cleave/post elution Chitin bead, 8: molecular weight marker. (**B**) Purified recombinant mouse apoA-II post HPLC purification. Lane 9: recombinant C57 apoA-II, 10: recombinant FVB apoA-II. ApoA-II-Intein-Chitin binding domain = 37.6 kd, Intein-Chitin binding domain = 28.7 kd, apoA-II = 9.0 kd.

Purification of C57 and FVB apoA-II was done using a method modified from Smith et al [21]. The human apoA-II-Intein-Chitin binding domain pTWIN1 construct was a generous gift from Dr. W. Sean Davidson (University of Cincinnati, Cincinnati, OH) [21]. Mouse apoA-II was generated by PCR of mouse liver cDNA and cloned into pTXB1 vector (New England Biolabs) using Nde1 and Sap1 sites. The mouse apoA-II sequence was cut from this construct using Nde1 and Age1 sites and inserted into the pTWIN1 construct, replacing the human apoA-II sequence. This construct was transformed into ER2566 cells (New England Biolabs). 1 L 2X YTA (+50 ug/ml ampicillin) was inoculated with 5 ml overnight culture and induced at 37°C with IPTG at OD600 = 0.6. After 3 hr expression cells were pelleted and resuspended in buffer A (8M Urea-PBS pH 7.5). Cells were disrupted 2X in a French press at 1200 psi and the lysate centrifuged at 15000× g for 30 min. Supernatant was diluted 1∶4 with PBS (final urea conc. = 2M) and bound on a column containing 7.5 ml washed Chitin beads (New England Biolabs) for 3 hrs at room temp or overnight at 4°C. Unbound protein was eluted and the beads washed 10× with 5 volumes of 2M Urea-PBS followed by 5–10 washes with 5 volumes of 1M Urea-PBS until the eluate was free of protein as measured by the BioRad protein assay (BioRad; Hercules, CA). Beads were resuspended in 2 bead volumes of 1M Urea-PBS +50 mM DTT and rotated 18 hrs at room temp. DTT cleaved sample was eluted from beads and another 2 volumes of 1M Urea-PBS +50 mM DTT added to beads and rotated at room temperature for 18 hrs. The sample was eluted from beads and the beads were washed with another 2 volumes of 1M Urea-PBS. All eluates were combined, and further purified on HPLC using a reverse-phase, C18 preparative HPLC column (Rainin Dynamax) with an acetonitrile gradient (5 min 25–45%, 20 min 45–49%, 1 min 49–100%, 10 min 100%) in 0.1% trifluoroacetic acid at room temperature. The apoA-II peak (19 min) was collected and lyophilized. Protein purity was greater than 95% by SDS PAGE (**Figure 1**). Samples were resuspended in 8M Urea-PBS and stored at −20°C.

### Circular Dichroism

C57 and FVB apoA-II protein stocks were dialyzed against PBS and protein concentration measured using the microBCA protein assay (Pierce; Rockford, IL). Circular dichroism was done on 300 µl protein stocks at concentrations between 225 and 250 µg/ml in a 0.1 cm cell using an AVIV Circular Dichroism Spectrometer, model 202. Triplicate spectra were taken using scans from 260 to 180 nm at 20 nm/min with a bandwidth of 1 nm and a slit width of 0.57 mm. Absorbance in millidegrees was converted to molar ellipticity using the molecular weight of each apoA-II variant. CD spectra were fitted using Olis GlobalWorks software with the Continll algorithm used to to derive protein secondary structure values [22].

### DMPC clearance

Dimyristoyl-phosphatidylcholine powder (DMPC; Avanti Polar Lipids, Alabaster, AL) was dissolved in chloroform and dried under airflow while vortexing. The DMPC film was resuspended in PBS at 1 mg/ml and vortexed vigorously for 1 minute to prepare multi-lamellar vesicles. DMPC multi-lamellar vesicles (0.25 mg/ml final concentration) were added to each well of a 96-well plate containing a final concentration range of 0.0–0.1 mg/ml of each recombinant protein. The decrease in absorbance over time at OD490 was measured every 30 sec for 20 min on a BioTek MicroQuant microplate reader and subtracted from a DMPC only (no protein control).

### ApoA-II deficient HDL isolation and biotinylation

The affinity of recombinant C57 and FVB apoA-II proteins for HDL was also measured using Surface Plasmon Resonance (SPR) on a BIAcore 3000 biosensor. HDL was purified from apoA-II deficient mice on the C57 background (Jackson Labs, Bar Harbor, ME). Mouse plasma was isolated by density gradient centrifugation. Plasma was brought to a density of 1.16 using NaBr to a final volume of 4 ml on top of which was layered 4 ml each of 1.125 and 1.063 NaBr density solutions. Density gradients were centrifuged for 17 hrs at 178,000× g and fractionated into 16 equal fractions. HDL fractions (by enzymatic cholesterol analysis, Roche Diagnostics, Indianapolis, IN) were combined and brought to 6 ml 1.25 density with NaBr and layered under 1.21 density solution and re-centrifuged. Purified apoA-II deficient HDL was then dialyzed/concentrated into PBS using a 100 kd cut-off Centricon concentrator (Millipore, Billerica, MA) and stored at 4°C. HDL protein concentration was determined by BioRad protein assay (Hercules, CA). *Apoa2^−/−^* HDL was biotinylated by incubating 0.5 mg HDL with a 100 fold molar excess of EZ-Link NHS-PEG4-Biotin (Pierce, Rockford, IL) for 4 hours on ice followed by dialysis into PBS. HDL biotinylation was 50% by avidin-agarose pull down (Pierce, Rockford, IL).

### ApoA-II binding to apoA-II deficient HDL

Biotinylated apoA-II deficient HDL was coupled to a Strepavidin SA sensor chip (Biacore Uppsala, Sweden). Prior to HDL immobilization, the chip surface was conditioned by 100 µl injection of 1M NaCl in 50 mM NaOH at a flow rate of 20 µl/min. HDL was immobilized by injecting 24 µg biotinylated HDL over the chip at a flow rate of 20 µl/min for 3 min until 2000 response units of HDL were bound to the chip surface, after which the chip was washed with PBS. The recombinant apoA-II analyte in PBS was injected at 25°C at concentrations ranging from 50 nM to 1500 nM at a flow rate of 20 µl/min for 1 min with a dissociation time of 1 min. No reference cell was used since apoA-II bound more to the free chip surface than to the immobilized HDL, a result also observed with apoA-I [23]. The association (Ka) and dissociation (Kd) constants were determined using BiaCore BIAevaluation software using a global fit of 1∶1 binding with drifting baseline.

### J774 cholesterol efflux

[^3^H]-cholesterol efflux from J774 cells (J774A.1 cell line, American Type Culture Collection, Manassas, VA) was performed as described previously [24] with the following modifications. J774 cells were loaded overnight with DMEM+1% FBS+ 25 µg/ml Acetylated Low Density Lipoprotein (LDL)+2 µg/ml Sandoz Acyl-CoA Cholesterol Acyltransferase (ACAT) inhibitor+1 µCi/ml [^3^H]-cholesterol in the presence of 0.3 mM 8-(4-chlorophenyl-thio)-cAMP (CPT-cAMP). The next day, the cells were washed 3X with PBS and DMEM containing varying amounts of recombinant apoA-II was added. After 4 hours the media was removed from the cells and media and cells were extracted and counted as described previously [24].

ATP Binding Cassette transporter (ABC) A1 and ABCG1-specific [^3^H]-cholesterol efflux was done using mock, ABCA1, or ABCG1 stably transfected BHK cells under a mifepristone-inducible switch which were a generous gift from Dr. Chongren Tang (University of Washington, Seattle, WA) [25], [26]. Mock, ABCA1, or ABCG1 transfected BHK cells were loaded overnight with DMEM+10% FBS+1 µCi/ml [^3^H]-cholesterol. 72 hrs later, cells were washed 3X with DMEM and induced with DMEM+1 mg/ml BSA+/−20 mM Mifepristone. After 24 hrs DMEM+/−20 mM Mifepristone containing varying amounts of recombinant apoA-II were added. After 4 hours the media was removed from the cells and media and cells were extracted and counted as described previously [24].

### Liver and Plasma apoA-II analysis

RNA was prepared from mouse liver using a Trizol extraction method (Invitrogen) and cDNA was prepared from 1–4 µg RNA using Superscript III (Invitrogen) according to the manufacturer's protocol. 2 µl of 1∶50 diluted cDNA was subjected to real-time PCR using SYBR green mix (SA Biosciences) and primer mixes (SA Biosciences). Gene expression was normalized to liver Hypoxanthine-guanine phosphoribosyltransferase (HPRT) or Glyceraldehyde 3-phosphate dehydrogenase (GAPDH) expression levels.

Plasma was collected from each mouse genetic background by isofluorane anesthesia and retro-orbital bleed. Plasma apoA-II levels were assessed by 14% SDS-PAGE western blot using a rabbit-anti-mouse apoA-II antibody which was a generous gift from Dr. Keiichi Higuchi (Shinshu University, Matsumoto, Japan) [20]. The Western blot signal was quantified using FluorChem v2.0 Spot Denso software.

Lipoproteins in FEA plasma were fractionated by FPLC and individual fractions were analyzed for cholesterol by a colorimetric enzyme assay kit (Roche Diagnostics, Indianapolis, IN). The same fractions were analyzed for apoA-II signal by western blot as above.

### Statistical Analysis

Results are presented as mean +/− standard deviation. Statistical differences were analyzed using two tailed Student's t-test. P<0.05 was considered significant.

## Results

Using a protocol modified from Smith and colleagues we were able to purify more than 2 mg mouse apoA-II recombinant protein per liter of starting culture (**Figure 1**) [21]. To our knowledge ours is the first reported expression of recombinant murine apoA-II. The reported yields of human apoA-II are nearly 10 mg/L culture. The murine apoA-II may have lower expression due to its more lipophilic nature. Additionally, while Smith et al were able to remove the post-DTT cleavage fusion protein by means of the attached His6 tag, our attempts at this were not successful. Using HPLC we were able to achieve a highly purified protein for both C57 and FVB apoA-II (**Figure 1B**).

**Figure 2:**
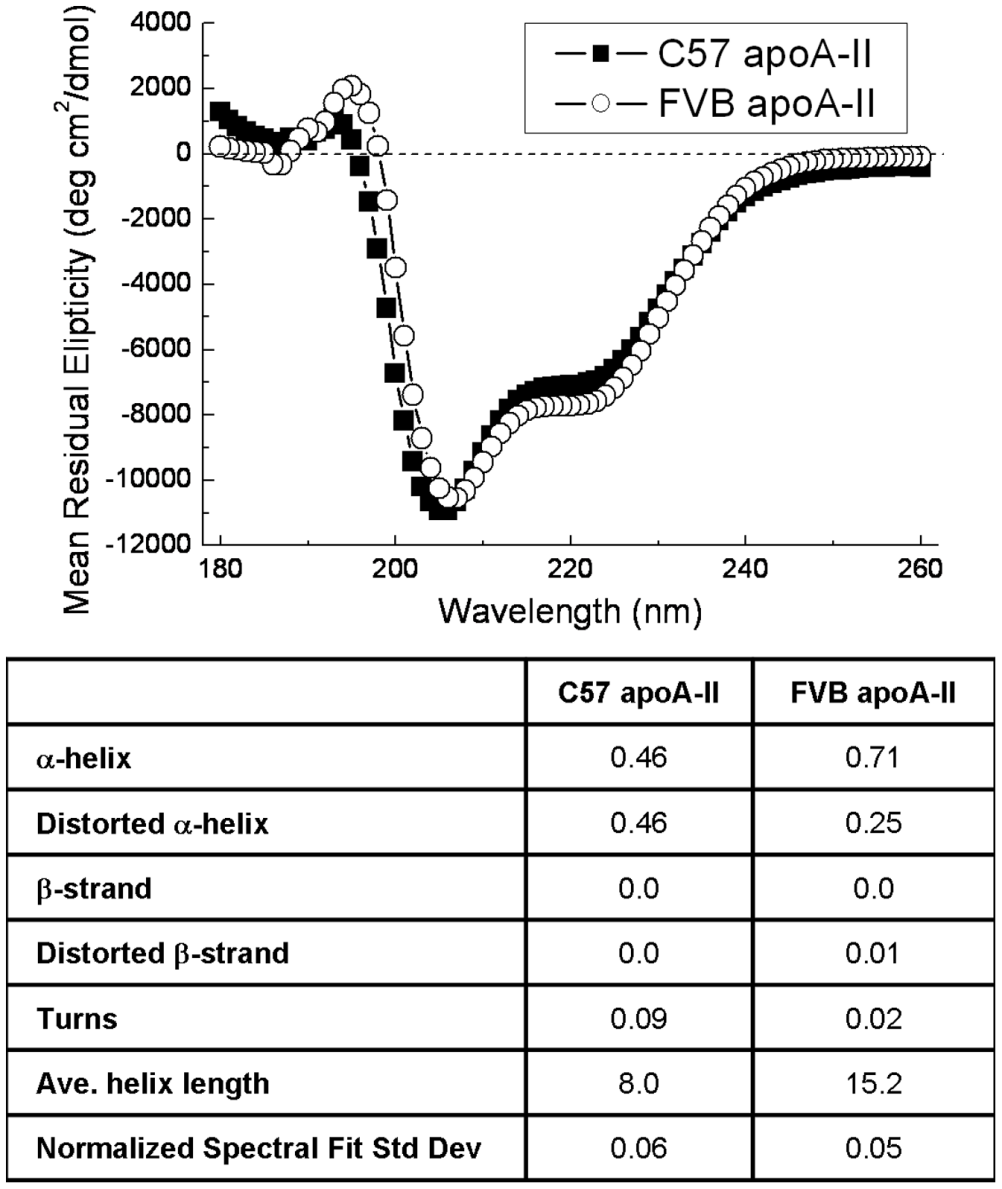
Circular dichroism of recombinant mouse apoA-II. Three accumulations of each sample were run at room temperature and the spectra shown as the overall mean. CD spectra were fitted using a Continll protein fit to derive protein secondary structure values.

The physical and functional nature of the murine apoA-II variants was assessed in several ways. First, the secondary structure of the protein was analyzed by Circular Dichroism (**Figure 2**). The fitted data indicates that the three amino acid changes in the FVB apoA-II alter the alpha-helicity of the protein. The total helicity (% alpha helix+% distorted alpha helix) of the protein is greater than 90% for both proteins. The alpha helicity of the FVB apoA-II is significantly higher than the C57 (71% vs 46%, respectively), and also higher than the 38–40% alpha-helicity reported for the recombinant human apoA-II protein [21]. The C57 apoA-II however has more distorted alpha helix than the FVB (46% for C57 vs 25% for the FVB). The average helical length of the FVB apoA-II was almost twice that of the C57 apoA-II, with the FVB apoA-II having fewer helix-disrupting turns. There was no significant β-strand in either protein.

**Figure 3:**
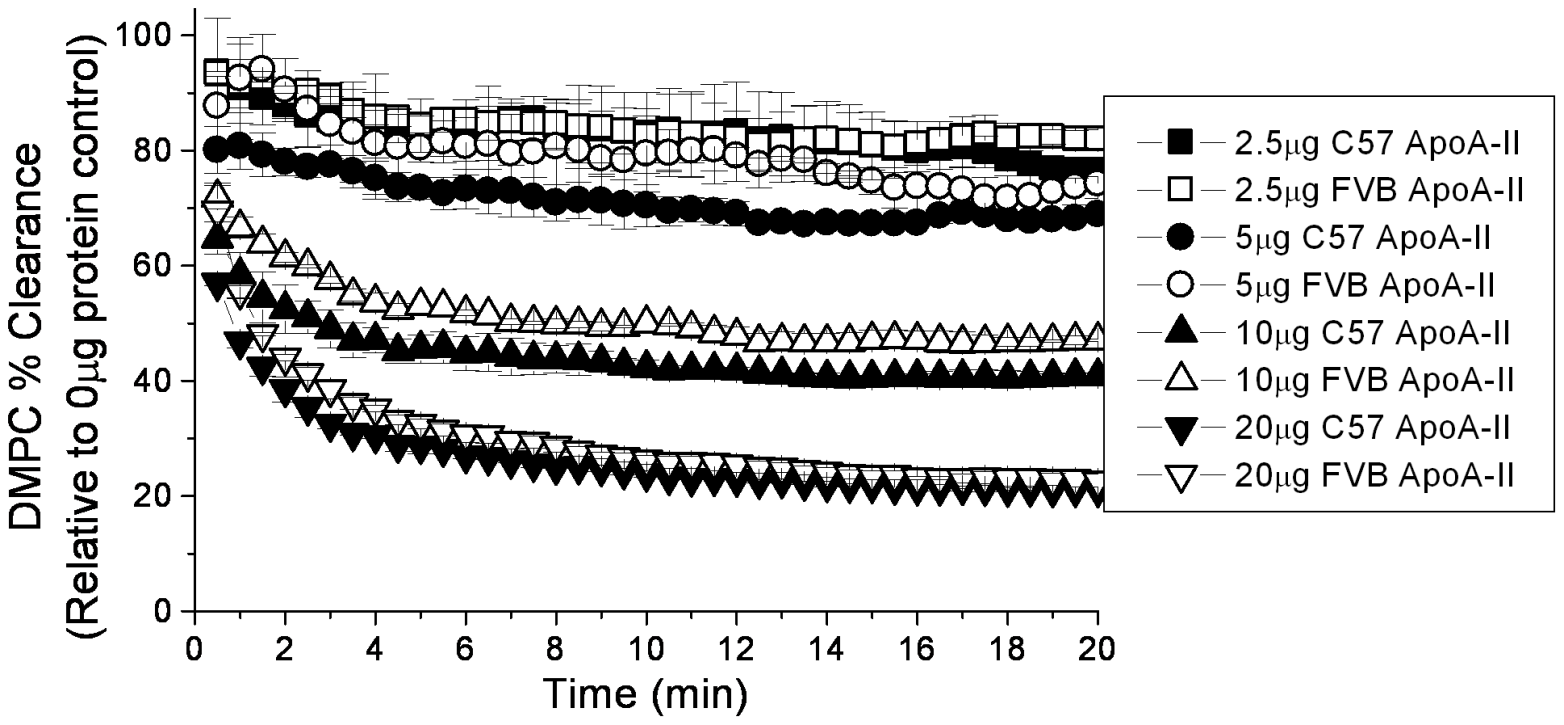
DMPC vesicle clearance by mouse apoA-II. DMPC multi-lamellar vesicles (0.25 mg/ml final concentration) were incubated at 25°C with 0.0–0.1 mg/ml of each recombinant protein (n = 2–3). The decrease in absorbance over time at OD490 was measured every 30(no protein control). Data represents mean +/− standard deviation.

The ability of a protein to clear dimyristoyl phosphatidylcholine (DMPC) micelles from solution has been a widely used measure of lipid binding, especially of apolipoproteins [27]–[29]. There was a small but consistent trend of more rapid clearance of DMPC micelles by C57 apoA-II (**Figure 3**). As the concentration of apoA-II increased, the difference in the amount of cleared micelle was only significant at the earliest time points (<1 min) and overall the lipid clearance was not significantly different. This data suggests that while the 3 amino acid variation between the C57 and FVB variants may alter the lipid binding capability of the protein, the difference is not dramatic.

**Figure 4:**
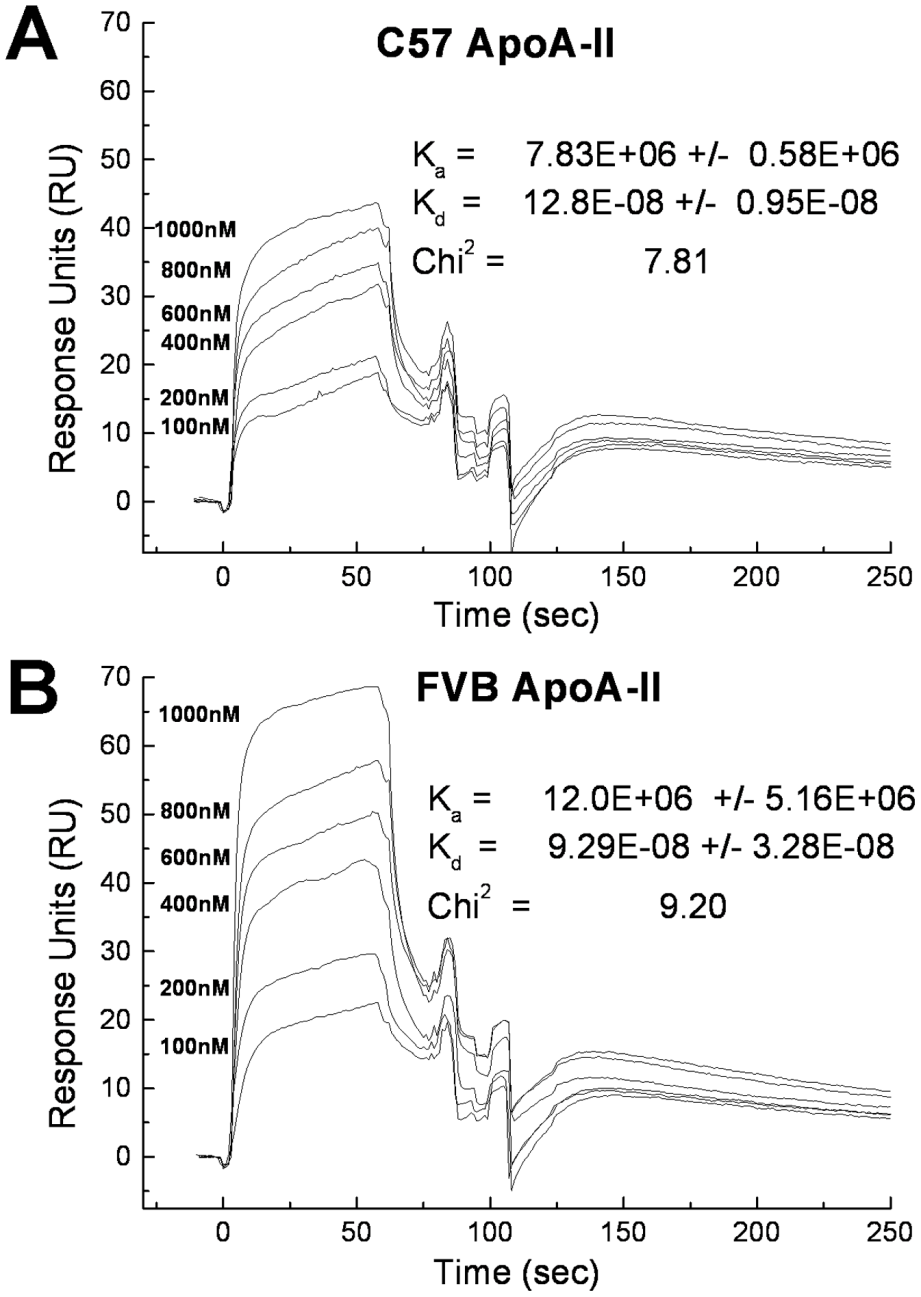
ApoA-II binding to apoA-II deficient HDL by Surface Plasmon Resonance. Biotinylated HDL isolated from *Apoa2^−/−^* mice was coupled to a Strepavidin SA sensor chip. The recombinant apoA-II analyte in PBS was injected at 25°C at concentrations ranging from 100 nM to 1000 nM at a flow rate of 20 µl/min for 1 min with a dissociation time of 1 min. The association (Ka) and dissociation (Kd) constants were determined using BiaCore BIAevaluation software using a global fit of 1∶1 binding with drifting baseline. Data shows mean +/− standard deviation for triplicate samples.

In wild type mice, the majority of circulating apoA-II resides on HDL. Using Surface Plasmon Resonance (SPR) the affinity of the two apoA-II variants for apoA-II deficient mouse HDL was assessed (**Figure 4**). No significant differences were noted in the association (K_a_) or dissociation (K_d_) constants of the two proteins.

**Figure 5:**
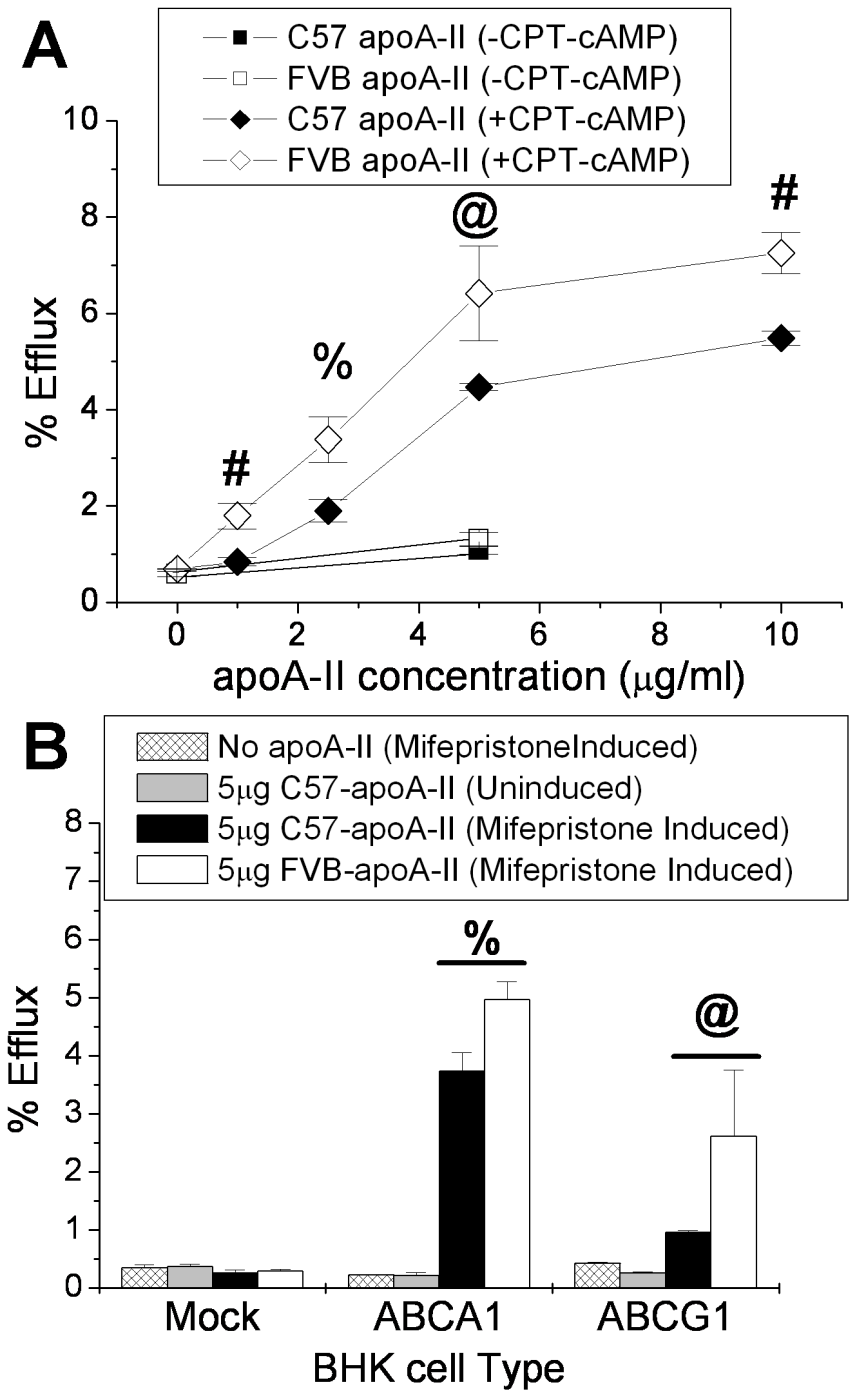
ApoA-II as an acceptor for cholesterol efflux from (A) mouse J774 macrophages, and (B) ABCA1- and ABCG1- overexpressing BHK cells. (**A**) J774 cells were incubated 24 hrs with 25 µg/ml Acetylated LDL+2 µg/ml Sandoz ACAT inhibitor+1 µCi/ml [^3^H]-cholesterol +/− the presence of 0.3 mM 8-(4-chlorophenyl-thio)-cAMP (CPT-cAMP). Cells were washed and incubated 4 hrs with varying concentrations of C57 or FVB apoA-II and the [^3^H]-cholesterol counted in the media and cells. (**B**) Mock, ABCA1, or ABCG1 transfected BHK cells were loaded overnight with 1 µCi/ml [^3^H]-cholesterol. Cells were washed and induced 24 hrs with 1 mg/ml BSA +/− 20 mM Mifepristone. Cells were washed and incubated 4 hrs with 0 or 5 µg of C57 or FVB apoA-II and the [^3^H]-cholesterol counted in the media and cells. Data represents mean +/− standard deviation for triplicate samples. @, p<0.05;%, p<0.01; #, p<0.005.

Reverse cholesterol transport is the process by which cholesterol is removed from peripheral macrophages and delivered by HDL to the liver for excretion as bile acids [30]. ApoA-I is the main player in this process by means of its interaction with the ABC transporters ABCA1 and ABCG1 on the cell surface of the macrophage, however human apoA-II has been shown to mediate this process as well [21]. We compared the ability of the two apoA-II mouse variants to act as acceptors for macrophage cholesterol efflux using the J774 mouse macrophage cell line in the presence or absence of CPT-cAMP which acts as an inducer of the ABC transporters (**Figure 5A**). In the absence of the cAMP, there is a tendency for FVB apoA-II to efflux more cholesterol than C57 apoA-II, but the efflux rate is so close to baseline that the difference is not significant. In the presence of cAMP induction, the FVB apoA-II mediated efflux is significantly greater than for C57 apoA-II at every concentration tested. The individual contribution of ABCA1 and ABCG1 to apoA-II mediated cholesterol efflux was examined using BHK cells which express ABCA1 or ABCG1 under a mifepristone inducible promoter [25], [26]. No efflux occurred in the absence of the transporters. The FVB apoA-II promoted significantly more cholesterol efflux than C57 apoA-II by means of both the ABCA1 and ABCG1 transporters (**Figure 5B**).

**Figure 6:**
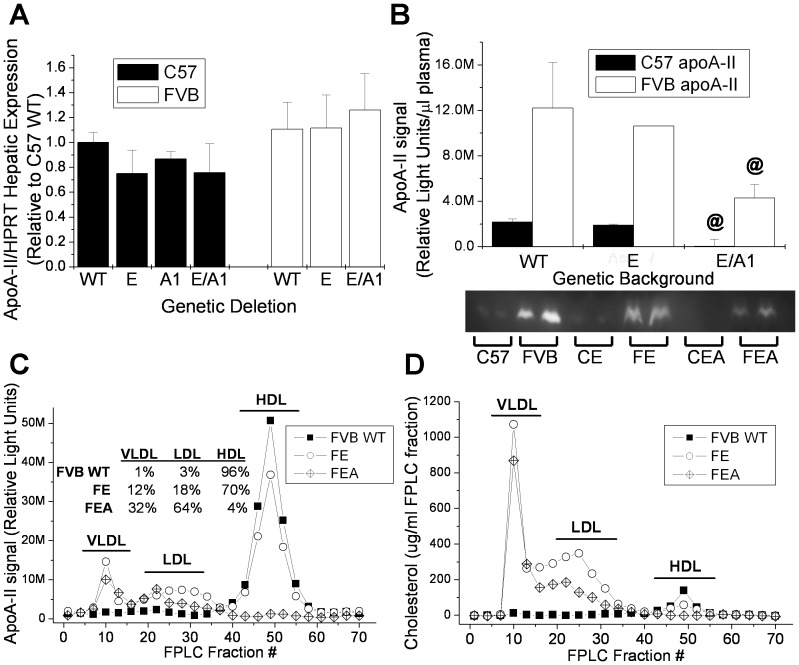
ApoA-II hepatic expression and plasma levels in wild type, *Apoe* knockout, and *Apoa1/Apoe* double knockout C57 and FVB mice. (**A**) ApoA-II hepatic RNA expression by real-time PCR. (**B**) Plasma apoA-II by western blot. @, p<0.05; apoE−/− vs EA−/−. Data represents mean +/− standard deviation for 2–5 samples. (**C**) FPLC fractionated plasma apoA-II by western blot along with percent of total apoA-II associated with each fraction. (**D**) FPLC fractionated plasma cholesterol.

The ability of murine apoA-II to mediate cholesterol efflux suggests that it may be able to form an HDL particle in the absence of other HDL-forming apoproteins such as apoA-I or apoE. We have made double knockout mice on the C57 and FVB backgrounds that lack both apoA-I and apoE and as a result lack a detectable HDL particle [31]. We have examined the effects of these deletions on apoA-II expression in both mouse strain backgrounds. As previously published by Doolittle et al, mice having either the C57 or FVB apoA-II sequence variations have similar apoA-II RNA hepatic expression levels (**Figure 6A**) [19]. Our results demonstrate the lack of apoE, apoA-I, or both proteins has no effect on *Apoa2* liver expression in either strain, nor was there a significant difference between the strains. This is in contrast to the results of Wang et al who indicated that apoA-I deficiency resulted in a small but significant age dependent increase in *Apoa2* liver [20]. At 2 months of age, similar to the age of the mice used in this study, they found *Apoa2* levels were 20% higher and 50% higher at 6 months. In order to be certain that the conflicting results were not due to differences in housekeeping genes (HPRT vs GAPDH) used in the qPCR assays in the two laboratories, we also checked *Apoa2* liver expression using GAPDH as the housekeeping gene. Our results were the same regardless of whether HPRT or GAPDH were used (data not shown). One possible explanation for the difference in results between ours and their liver expression data may be due to dietary effects, as our diet was 10% higher in total fat. Several studies have shown evidence that dietary fat composition impacts impacts apolipoprotein expression and this merits further investigation [32], [33].

Relative apoA-II plasma protein levels were compared among the knockout strains by western blot. As reported previously by our lab, apoA-II plasma levels are significantly higher in the wild type FVB strain than in the C57 despite similar RNA expression (**Figure 6B**) [17]. The lack of apoE has no effect on apoA-II levels in either strain. The *Apoa1/Apoe* double knockout results in FVB plasma apoA-II levels that are less than half that of the wild type levels. ApoA-II is undetectable in the C57 double knockout. FPLC fractions of the FVB WT, *Apoe^−/−^* (FE), and *Apoa1/Apoe* double knockout (FEA) were probed by western blot for apoA-II and for cholesterol to identify lipoproteins. As expected, in the wild type mouse which has a lipoprotein profile made up almost entirely of HDL, the apoA-II is found almost exclusively in the HDL fractions (**Figure 6C and D**). The absence of apoE results in a small fraction of the total apoA-II associating with the Very Low Density Lipoprotein (VLDL) and LDL fractions, but most is still found in association with the HDL fractions. In the absence of both apoA-I and apoE, most of the apoA-II is found on the larger VLDL and LDL particles where all of the plasma cholesterol is found. However, we do find that FVB apoA-II is also able to form an HDL-sized particle in the absence of apoA-I and apoE (4% of total plasma apoA-II).

## Discussion

ApoA-II is the second most abundant protein on HDL but its functions are only beginning to be understood. Until recently, the purification of large amounts of apoA-II was difficult but new methodology has enabled bacterial expression of milligram quantities of human apoA-II [21], [34]. Our modification of this protocol has allowed for the purification of multiple naturally-occurring variants of murine apoA-II and the comparison of their physical nature and function. The change in helicity of the proteins when the FVB mutations are introduced suggested that the protein-lipid interactions might be altered. However no significant differences in lipid or HDL binding were noted. The differences in helicity may explain the altered interaction of the apoA-II variants with the ABC transporters in promoting cholesterol efflux as altering the helices of apoA-I affect that protein's ability to mediate ABCA1 cholesterol efflux [35]–[37]. The disconnect between lipid/HDL binding ability and ability to promote ABCA1/ABCG1-mediated cellular cholesterol efflux is not surprising. We have previously reported on C57 and FVB apoA-I differences in HDL binding, yet with no significant differences in their ability to act as cellular cholesterol acceptors [17].

Our results using the murine apoA-II as an acceptor for ABCG1 mediated efflux were somewhat unexpected. Lipid free mouse and human apoA-I has been shown to act as an excellent acceptor for ABCA1-mediated cholesterol efflux, as has human apoA-II [21], [35]. Lipid free apoA-I and human apoA-II have both been shown to be essentially incapable of mediating cholesterol efflux by means of ABCG1 [38], while both apoA-I and apoA-II lipid discs are able to act as ABCG1 cholesterol acceptors, with apoA-II discs being the more effective of the two [26]. Additionally, the concentration of both apoA-I and apoA-II on HDL is correlated with its ability to efflux cholesterol by means of ABCG1 [26]. Based on these results with lipid free human apoA-II, we expected little ABCG1-mediated cholesterol efflux with our lipid-free murine apoA-II proteins, and were surprised to discover that both were quite effective, although the FVB apoA-II was significantly better. What this means physiologically is not completely clear. ApoA-II status is dependent on the presence of HDL, as evidenced by the reduced plasma levels of apoA-II in FVB *Apoa1/Apoe* double knockout mice and the complete disappearance of plasma apoA-II in the C57 double knockouts. The ability of the apoA-II variants to interact with both ABCA1 and ABCG1 to form an apoA-II HDL particle independent of apoA-I may be important in the observed differences in plasma HDL cholesterol levels between strains possessing the two apoA-II variants. Previous results from our laboratory have shown no difference in ABCG1-mediated cholesterol efflux between C57 and FVB HDL despite FVB HDL possessing twice the amount of apoA-II as C57 HDL [17]. It may be that lipid-free and nascent apoA-II HDL functions differently than the mature apoA-I/apoA-II HDL particle with respect to its ABC transporter interactions. Other apoproteins may also be impacting apoA-II function. In the absence of apoA-I alone, apoA-II redistributes to a larger HDL in an age related manner [20]. However, our results show that in the *Apoa1/Apoe* double deficient FVB mice the majority of plasma apoA-II is found on the larger VLDL and LDL particles, demonstrating the wide range of lipoprotein particle size with which apoA-II can associate, but suggests a preference for HDL particles when the apoA-I or apoE is present.

While this study has dealt mainly with the lipid binding and cholesterol efflux differences between the apoA-II variants, there are numerous other facets of apoA-II function to be explored. ApoA-II has been shown to be inhibit the hydrolysis of HDL lipid by hepatic lipase [39]. The anti-inflammatory properties of HDL are also modified by apoA-II, particularly by the apoA-I/apoA-II ratio of the HDL [40]. Other variants of mouse apoA-II (apoA-II^c^) have been shown to be susceptible to amyloidosis [14] and deletion of apoA-I promotes amyloid formation even of the C57 apoA-II variant [20]. The findings presented here add to the growing body of data that indicate the functions of apoA-II as well as its interaction with other apoproteins may be modified by the single nucleotide polymorphisms that exist in inbred mouse strains.
